# Molecular Testing and Surgical Outcomes in Bethesda III and IV Thyroid Nodules: A Retrospective Cohort Study

**DOI:** 10.3390/cancers17203376

**Published:** 2025-10-20

**Authors:** Alexandra E. Payne, Layla Gobeil, Marc P. Pusztaszeri, Isabelle Bannister, Saruchi Bandargal, Sabrina Daniela da Silva, Veronique-Isabelle Forest

**Affiliations:** 1 Faculty of Arts and Sciences, Duke University, Durham, NC 27708, USA; 2 Faculty of Medicine, McGill University, Montreal, QC H3A 2M7, Canada; 3 Department of Pathology, Sir Mortimer B. Davis-Jewish General Hospital, McGill University, Montreal, QC H3T 1E2, Canada; marc.pusztaszeri@mcgill.ca; 4 Faculty of Arts and Sciences, Queens University, Kingston, ON K7L 3N6, Canada; 5 Department of Otolaryngology Head and Neck Surgery, Sir Mortimer B. Davis-Jewish General Hospital, McGill University, Montreal, QC H3T 1E2, Canada

**Keywords:** thyroid cancer, Bethesda system, molecular testing, *BRAF*, *RAS*, hemithyroidectomy, total thyroidectomy, indeterminate thyroid nodules

## Abstract

**Simple Summary:**

Thyroid nodules are frequently encountered in clinical practice, particularly among women. Although the majority are benign, indeterminate cytology remains a diagnostic challenge. Bethesda III and IV nodules represent categories with an uncertain risk of malignancy, often leading to unnecessary surgeries or suboptimal interventions. Molecular testing has emerged as a promising adjunct to refine risk stratification and tailor surgical management. This study investigated the role of molecular testing in guiding optimal treatment for Bethesda III and IV thyroid nodules, demonstrating its potential to reduce overtreatment and undertreatment and improve clinical outcomes.

**Abstract:**

**Background**: Bethesda III and IV thyroid nodules, which fall under the category of indeterminate cytology, pose challenges in clinical decision-making due to their ambiguous risk of malignancy. Molecular testing has been increasingly employed to aid risk stratification and optimize the extent of surgical intervention. **Methods**: A retrospective review of 410 patients with Bethesda III and IV thyroid nodules who underwent thyroid surgery at McGill University teaching hospitals between January 2016 and April 2022. Patients were grouped based on whether or not they underwent preoperative molecular testing. Data were collected on demographic variables, histopathologic diagnosis, mutation profiles, and surgical outcomes. The primary outcome was to assess for concordance between surgical intervention and final pathology in both groups, with a focus on identifying optimal versus suboptimal management. Optimal management is defined as surgery appropriate to the aggressiveness of disease, meaning a hemi-thyroidectomy for a non-aggressive malignancy, total thyroidectomy for an aggressive malignancy, and no surgery for a benign nodule. Furthermore, suboptimal management includes unnecessary surgery or incorrect surgery for the level of aggressivity of the nodule. **Results**: Among the 410 patients, 203 underwent molecular testing, while 207 did not. Of those who underwent molecular testing, 117 had Bethesda III nodules and 86 had Bethesda IV nodules. In the non-tested group, 129 and 78 patients had Bethesda III and IV nodules, respectively. Optimal surgical intervention was achieved in 67.5% of patients who underwent molecular testing, compared with 25.1% in those who did not (*p* < 0.001). Subgroup analysis revealed that 61.5% of Bethesda III nodules with molecular testing received optimal care versus 21.0% of those without testing. In the Bethesda IV cohort, optimal surgery was achieved in 75.6% with testing versus 32.1% without. Among the suboptimally managed patients, 70.1% (155/221) were from the group that did not undergo molecular testing. In addition, molecular testing identified aggressive mutations such as *BRAF V600E* and *TERT* promoter mutations more frequently in Bethesda III nodules, while *RAS*-like mutations, associated with indolent behavior, predominated in Bethesda IV nodules. **Conclusions**: In this study, molecular testing significantly improved risk stratification and the likelihood of optimal surgical management in patients with Bethesda III and IV thyroid nodules. Incorporating molecular diagnostics into the standard preoperative workflow may enhance patient care, reduce unnecessary surgeries, and optimize the extent of surgery. Future studies should evaluate the cost-effectiveness and broader implementation of molecular testing in diverse healthcare settings.

## 1. Introduction

Thyroid nodules are a common clinical finding, with a prevalence ranging from 19% to 68% among randomly selected individuals when assessed by high-resolution ultrasound [1]. In the U.S., they are about four times more prevalent in women than in men [2]. While most nodules are benign, differentiating benign from malignant nodules remains a cornerstone of thyroid nodule management [3,4]. Fine needle aspiration (FNA) biopsy, guided by ultrasound, is the gold standard for evaluating thyroid nodules, and its results are categorized using the Bethesda System for Reporting Thyroid Cytopathology (TBSRTC) [5,6].

Bethesda categories range from I (nondiagnostic) to VI (malignant), with Bethesda III (atypia of undetermined significance or follicular lesion of undetermined significance) and Bethesda IV (follicular neoplasm or suspicious for a follicular neoplasm) representing indeterminate diagnoses [7,8]. The malignancy risks for these categories vary, but the latest edition of the Bethesda system estimates them to be ~22% for Bethesda III and ~30% for Bethesda IV [9]. However, these estimates are known to differ significantly between institutions and populations [6,9]. Because of this ambiguity, patients with Bethesda III and IV nodules have often undergone diagnostic surgery, leading to unnecessary procedures in many cases where pathology later reveals benign disease [10,11].

Molecular testing has become increasingly integrated into the preoperative assessment process. These tests can detect alterations that correlate with tumor behavior [12]. Commonly evaluated mutations include *BRAF*, *RAS* (*HRAS*, *KRAS*, *NRAS*), *TERT* promoter mutations, *RET*/*PTC* rearrangements, and *TP53*. *RAS*-like mutations are generally associated with indolent tumors that exhibit a low risk of capsular or vascular invasion and can often be managed with a hemithyroidectomy, while *BRAF*-like mutations often indicate aggressive phenotypes, higher risk of extrathyroidal extension or nodal metastasis, and typically warrant a total thyroidectomy [13].

Several major societies now acknowledge the role of molecular testing in Bethesda III and IV nodules. The 2025 American Thyroid Association (ATA) guidelines recommend that molecular diagnostics may be used as an adjunct to refine malignancy risk in indeterminate nodules and guide surgical decision-making [14]. European Thyroid Association guidelines and NCCN guidelines similarly support the use of molecular testing in this setting, although they emphasize that testing should complement, rather than replace, clinical and sonographic assessment [15,16].

Beyond surgical intervention, alternative management strategies for indeterminate nodules include repeat FNA or core-needle biopsy, active surveillance in selected low-risk patients, and minimally invasive approaches such as radiofrequency or ethanol ablation [17]. However, for Bethesda III and IV nodules, these approaches have limitations: repeat biopsies often yield persistent indeterminate results, active surveillance carries uncertainty given the 22–30% malignancy risk, and minimally invasive ablation does not provide a surgical specimen for definitive histopathology [9]. Thus, while considered in the guidelines, their adoption for Bethesda III/Bethesda IV nodules remains cautious and highly dependent on local expertise and patient preference.

This study aimed to assess whether the implementation of molecular testing in Bethesda III and IV nodules contributes to improved clinical decision-making and optimized surgical intervention. By analyzing a large cohort of patients at a tertiary care center, we sought to determine the relationship between molecular profiles and surgical outcomes.

## 2. Materials and Methods

### 2.1. Study Design and Patient Selection

A retrospective chart review was conducted on patients who underwent thyroid surgery for cytologically indeterminate nodules (Bethesda III or IV). There were 410 cases of patients undergoing surgery from January 2016 to April 2022 at McGill University Teaching Hospitals in Montreal, Canada including the Jewish General Hospital and the McGill University Health Center. All eligible Bethesda III and IV cases during the study period were included consecutively, and the use of molecular testing was decided according to standardized institutional criteria rather than individual physician preference. This approach helps reduce selection bias by ensuring that testing decisions are consistent and not influenced by patient characteristics or clinician judgment.

Institutional ethical approval was obtained, and patient consent was waived due to the study’s retrospective nature. This study was accepted by the Medical/Biomedical Research Ethics Committee (REC) of CIUSSS West-Central Montreal Research Ethics Board (REB) (#2024-4062).

The inclusion criteria were as follows: adult patients (≥18 years old) with Bethesda III or IV nodules who had undergone thyroid surgery. Patients were stratified into two groups based on whether molecular testing was performed. Exclusion criteria included the presence of compressive symptoms, Graves’ disease, nodules > 4 cm, confirmed lymph node metastases on preoperative imaging, and concurrent parathyroid surgery.

All patients had complete demographic, cytology, surgical, and pathology data available in the electronic medical record. No patients were lost to follow-up or excluded due to incomplete data, and there were no protocol deviations.

### 2.2. Data Collection

Demographic data, cytology results, molecular testing reports, surgical procedures (hemi-thyroidectomy and total thyroidectomy), and the final pathology outcomes were collected. Molecular testing platforms used included ThyroSeq v3, ThyGenX, and Afirma GSC. Molecular testing was performed on residual cytology material from the diagnostic FNA sample; if insufficient, cell-block preparations were utilized. Each testing platform operates slightly differently. ThyroseqV3 uses next-generation sequencing, in order to detect point mutations and gene fusions as well as copy number alterations across more than 100 genes. ThyGenNEXT/ThyraMIR, on the other hand, combines targeted DNA genotyping with microRNA expression profiling to improve risk stratification. Finally, Affirma GSC uses RNA expression-based machine learning to differentiate benign from suspicious nodules [12]. The sensitivity and specificity reported for each test varies depending on the study, but an analysis conducted by Connelly et al. revealed that sensitivity and specificity were 87.5% and 53.3% for ThyroSeq, 66.7% and 48.3% for ThyGeNEXT/ThyraMIR, and 73.1% and 54.7% for Afirma, respectively [18]. Access to testing was not standardized by institutional protocol, rather, testing was ordered at the discretion of the treating physician and was influenced by patient preference, insurance coverage, and availability. As such, group allocation was determined by clinical practice patterns rather than random assignment. We acknowledge that socioeconomic factors and disparities in access to molecular testing may have contributed to differences between groups. Test results were used to identify the presence of specific mutations and classify the potential aggressiveness of the nodule. All three molecular platforms (ThyroSeq v3, ThyGenX, Afirma GSC) were available at our institution throughout the entire study period. We found no evidence of formal changes in institutional guidelines or platform preference over time. However, we cannot rule out unmeasured shifts in physician ordering habits or insurance coverage that might have influenced test selection.

### 2.3. Definition of Optimal Management

Surgical intervention was deemed optimal in the following scenarios: hemi-thyroidectomy for malignancy not warranting a total thyroidectomy. A total thyroidectomy was considered appropriate for invasive/aggressive malignancy, defined by established criteria from the American Thyroid Association and World Health Organization guidelines including tumor size > 4 cm, extrathyroidal extension, aggressive histological subtypes (hobnail, tall cell, columnar, diffuse sclerosing), extensive lymphovascular invasion, or lymph node metastasis [14,19]. Non-invasive follicular thyroid neoplasm with papillary-like nuclear features (NIFTP) was classified as an indolent neoplasm, for which hemithyroidectomy was considered an adequate and definitive surgical approach [14].

Suboptimal management was defined as unnecessary surgery for benign nodules, hemithyroidectomy for invasive/aggressive malignancies, and total thyroidectomy for indolent malignancies/non-invasive follicular thyroid neoplasm with papillary-like nuclear features (NIFTP).

### 2.4. Statistical Analysis

Descriptive statistics were used to summarize the patient’s demographics, nodule characteristics, and surgical outcomes. T-tests and Chi-squared tests were performed to compare categorical and continuous variables between groups with and without molecular testing. Statistical significance was set at *p* < 0.05.

Comparisons between groups were performed using *t*-tests and Chi-squared tests. We recognize that variables such as age, sex, tumor size, and comorbidities may influence surgical outcomes, however, due to the retrospective design and primary focus on concordance between intervention and pathology, no multivariable adjustments were performed.

### 2.5. Ethical Consideration

McGill University’s institutional review board authorized this study. Informed consent was waived due to the retrospective nature of the chart review. To ensure confidentiality, all patient data were anonymized. This techniques section describes the approach used to retrospectively assess the influence of molecular testing on the surgical management of Bethesda III and IV thyroid nodules, with the intention of determining the efficacy of molecular testing in improving surgical outcomes and minimizing unnecessary procedures.

## 3. Results

**Figure 1:**
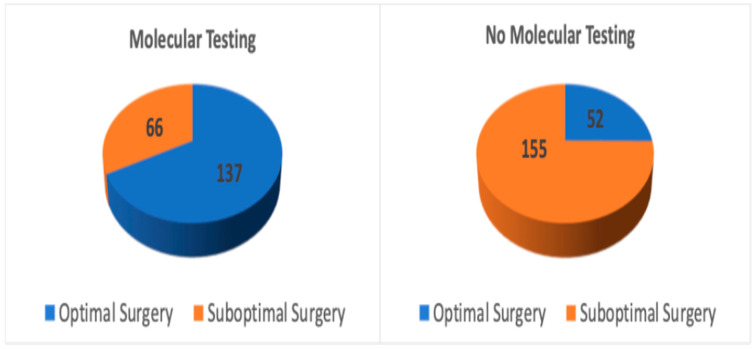
Surgical outcomes in all patients with vs. without molecular testing.

A total of 410 patients met the inclusion criteria. The mean age was 52.8 years (range: 16–88), with a female predominance (81.2%). In the overall cohort, 203 patients underwent molecular testing, while 207 patients did not. A total of 67.5% (137/203) of patients who underwent molecular testing received optimal surgical management, while 32.5% (66/203) did not. In contrast, 25.1% (52/207) of patients without molecular testing received optimal surgery, while 74.9% (155/207) did not (*p* < 0.001) (Table 1, Figure 1).

Among the 117 patients with Bethesda III nodules who underwent molecular testing, 61.5% (72/117) were identified as having received optimal surgical management. Of the 45 patients deemed to have received suboptimal treatment (38.5%), 4 underwent total thyroidectomies and 41 underwent hemi-thyroidectomies. Final pathology revealed that 87.8% (36/41) of hemi-thyroidectomies were for benign nodules. Among the 129 patients with Bethesda III nodules who did not undergo molecular testing, 20.9% (27/129) of patients were determined to have received optimal surgical care. Of the 102 patients deemed to have received suboptimal treatment, 30 underwent total thyroidectomies and 72 hemi-thyroidectomies. Final pathology revealed that 88.9% (64/72) of the hemithyroidectomies were performed for benign nodules (Table 2). The difference in optimal surgical outcomes between the tested and non-tested patients was statistically significant (*p* < 0.001).

Among the 86 patients with Bethesda IV nodules who underwent molecular testing, 75.6% (65/86) was identified as having received optimal surgical management. Of the 21 patients deemed to have received suboptimal treatment (24.4%), 1 underwent a total thyroidectomy and 20 underwent hemi-thyroidectomies. Final pathology revealed that 70% (14/20) of hemi-thyroidectomies were for benign nodules. Among the 78 patients with Bethesda IV nodules who did not undergo molecular testing, 32.1% (25/78) of patients were determined to have received optimal surgical care. Of the 53 patients deemed to have received suboptimal treatment (67.9%), 14 underwent total thyroidectomies and 39 underwent hemithyroidectomies. Final pathology revealed that 76.9% (30/39) of hemi-thyroidectomies were for benign nodules (Table 3). The difference in optimal surgical outcomes between the tested and non-tested patients was statistically significant (*p* < 0.001).

**Figure 2:**
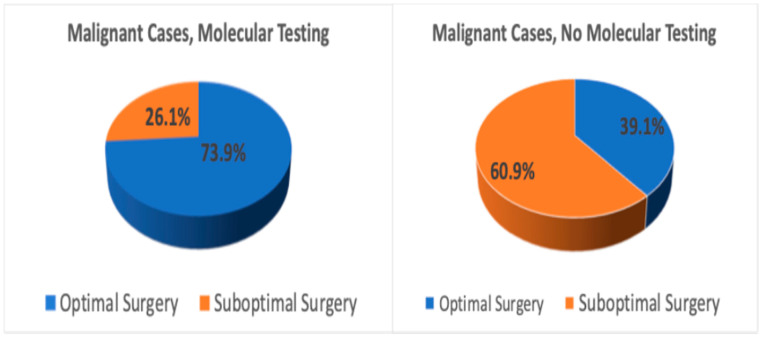
Surgical outcomes in malignant cases with vs. without molecular testing.

Further analysis showed that 73.9% of patients who underwent molecular testing and were later confirmed to have malignancy received optimal surgery. In contrast, 39.1% of malignant cases without molecular testing were managed optimally (Figure 2). Of the 221 patients who experienced suboptimal surgical outcomes, 70.1% (155/221) had not received molecular testing.

## 4. Discussion

This study highlights the significant impact of molecular testing on the optimal surgical management of indeterminate thyroid nodules (Bethesda III and IV). Optimal management is defined as selecting the appropriate surgical approach for malignant nodules and NIFTP, and avoiding surgery for benign disease. In this framework, benign nodules do not warrant surgery, non-aggressive malignancies and NIFTP should be treated with a hemi-thyroidectomy, and aggressive malignancies require a total thyroidectomy [14]. One of the primary drivers of outcome differences between the two groups was the high rate of unnecessary surgery (benign nodules) in the group that did not undergo molecular testing. Advances in molecular diagnostics have been proposed as an adjunct to cytology to improve preoperative risk stratification and reduce avoidable thyroidectomies [20]. Traditionally, diagnostic surgery has been performed in patients with indeterminate nodules [21], despite the majority of these nodules ultimately being non-malignant. Our study highlights the reduction in surgery for benign disease in patients with indeterminate thyroid nodules who undergo molecular testing. The findings reinforce the heterogeneity of indeterminate nodules and support the use of molecular testing to better characterize the aggressiveness/invasiveness of these nodules [22,23]. Identifying high-risk mutations preoperatively facilitates total thyroidectomy at the initial surgery, avoiding the need for a second operation. On the other hand, identifying indolent mutations or benign profiles helps avoid overtreatment. The difference in optimal surgery rate between the group undergoing molecular testing and the one that did not undergo molecular testing (67.5% vs. 25.1%, respectively) clearly demonstrates this point.

Avoiding unnecessary surgical treatment for benign or indolent nodules offers substantial benefits, as thyroid surgeries carry potential health risks and contribute to increased healthcare costs and system burden. Possible thyroid surgery complications include permanent or transient hypoparathyroidism, recurrent laryngeal nerve injury, and postoperative bleeding [24,25]. Additionally, overtreatment can also pose a threat to the psychological well-being and quality of life of patients, with higher rates of anxiety and depression reported, related to the risk of their cancer eventually coming back [26]. Beyond clinical implications, overtreatment carries a substantial financial burden. A recent study conducted across five hospitals in Quebec evaluated publicly funded ThyroSeq v3 testing in 500 patients with Bethesda III and IV thyroid nodules between November 2021 and November 2022. The report showed a 72.6% benign call rate, with 99.7% of test-negative patients avoiding surgery over a two-year follow-up. The cost-effectiveness analysis revealed that incorporating molecular testing into the public healthcare system could save approximately CAD 6.1 million over ten years by reducing unnecessary surgeries and associated complications [27]. In a pooled meta-analysis of 31 studies including 4464 indeterminate thyroid nodules, surgical avoidance rates varied across molecular testing platforms, ranging from 50.3% for ThyroSeq v2 to 68.6% for ThyGenX/ThyraMIR, with Afirma GSC and ThyroSeq v3 showing intermediate rates (50.6% and 62.5%, respectively). Such variation likely reflects differences in test design and validation [23]. Although multiple studies have looked at the overall cost-effectiveness of molecular testing, few have yet to compare the economic performances of specific tests. Further investigation should be conducted in the future. Beyond the clinical benefits, the cost-effectiveness of molecular testing is an important determinant in healthcare policy. Showing that these tests provide good value for money can support their inclusion in publicly funded programs and guide reimbursement decisions. These policies could make testing more accessible to patients, especially in settings where cost remains an important barrier [27]. Testing preoperatively also helps identify whether a total thyroidectomy is needed before the initial surgery, thus avoiding the need for a second operation, which carries a higher risk of complications. In fact, in a study by Medas et al., patients undergoing a second surgery experienced higher rates of transient hypoparathyroidism, permanent hypoparathyroidism, and recurrent laryngeal nerve damage than those undergoing only one [28]. Preoperative molecular testing enables the selection of the appropriate surgical approach from the outset, potentially reducing the need for a second surgery and its associated risks [21].

In this study, Bethesda III nodules, despite having a lower overall malignancy rate, were more likely to harbor aggressive mutations when malignant. This may be related to the fact that Bethesda III nodules are a much more heterogeneous group than Bethesda IV nodules, and limited/poor sampling often precludes a more definitive diagnosis in this group. This suggests a paradoxical pattern wherein the nodules with lower expected malignancy can behave more aggressively when cancer is present [29]. Bourque et al. analyzed 114 Bethesda III nodules and identified a diverse molecular profile, with *RAS* mutations in 34.2% of cases, CNA/GEP and EIF1AX alterations in 29.8%, and high-risk mutations, such as *BRAF V600E*, *TERT* promoter, or *TP53* mutations, in 8.8% of nodules, further supporting the potential aggressiveness within this category [30]. Bethesda III nodules are often characterized as low risk, as they are less likely to be malignant; however, their aggressive tendency is worth considering. A recent retrospective study showed that out of 628 Bethesda III and Bethesda IV nodules that were evaluated, 18.6% of Bethesda III nodules had aggressive characteristics compared with 10.2% for the Bethesda IV nodules [21]. Molecular testing can identify the presence of high-risk mutations such as *BRAF V600E*, which may indicate the need for a more aggressive surgical approach [31]. In a recent comparative cohort study, 217 patients with either Bethesda III or Bethesda IV nodules underwent molecular testing, and the most frequent mutations identified were analyzed. Bethesda IV nodules mostly presented *RAS-like* mutations, which are commonly associated with low rates of aggressive histopathological features and indolent behavior. On the other hand, *BRAF V600E* mutations, which are considered to be more aggressive, were found exclusively in Bethesda III and not in Bethesda IV nodules [32,33].

Bethesda IV nodules are more likely to be malignant but generally less aggressive. These nodules commonly feature *RAS-like* mutations and copy number alterations (CNAs) that are not strongly predictive of aggressive behavior. As a result, patients with Bethesda IV nodules may benefit from more conservative surgical approaches, especially when molecular testing supports a non-aggressive profile. For example, patients presenting with these types of nodules may be managed with a hemi-thyroidectomy rather than a total thyroidectomy, the latter being associated with greater risks and potential long-term consequences [34]. Unlike those who undergo a total thyroidectomy, patients treated with a hemi-thyroidectomy retain a functional portion of their thyroid gland and may not require lifelong thyroid hormone replacement therapy [35]. Preoperative molecular testing can help identify genetic markers associated with less aggressive cancer behaviors, allowing clinicians to safely opt for more conservative surgical approaches, thus avoiding the multiple possible negative side effects associated with a thyroidectomy.

Our data support the current literature suggesting that molecular testing should be considered a standard part of preoperative evaluation in indeterminate thyroid nodules [29]. However, important barriers to widespread adoption exist, which include high costs, limited access in some regions, and a lack of physician experience and education. Expanding insurance coverage, integrating cost-effective platforms, and improved educational resources for physicians could increase accessibility. Future studies incorporating long-term surveillance, such as recurrence, survival, and patient-reported outcomes, are needed to confirm that early surgical decisions aided by molecular testing translate into durable oncologic and quality-of-life benefits.

Selection bias cannot be entirely ruled out, as this was a retrospective, single-center study. However, the inclusion of all eligible Bethesda III and IV cases during the study period helps minimize this risk. Additional limitations include the retrospective nature of the study, the use of multiple molecular testing platforms, and potential differences in socioeconomic or clinical characteristics between patients who underwent molecular testing and those who did not. Although the study was conducted at a single tertiary-care institution, our patient population and clinical approach were comparable to those seen in other large academic hospitals, suggesting that the findings are likely applicable beyond our institution. Nonetheless, validation in larger, multicenter cohorts would be important to confirm these results and ensure their generalizability across different healthcare settings and patient demographics. Because inclusion criteria excluded nodules larger than 4 cm as well as fixed or metastatic lesions, the findings primarily apply to smaller, non-fixed, non-metastatic nodules, which limits generalizability to other clinical presentations.

While our results support the value of molecular testing, test performance and applicability may vary depending on local disease prevalence, mutation spectrum, and healthcare accessibility, emphasizing the need for context-specific implementation strategies. Additionally, as our analysis was univariate and did not adjust for baseline variables such as age, nodule size, or evolving practice patterns, residual confounding cannot be excluded. Finally, our study was not designed to evaluate specific mutation profiles, cost-effectiveness, or postoperative outcomes. These important questions warrant investigation in future prospective, multicenter studies.

## 5. Conclusions

This study shows that molecular testing was associated with improved surgical decision-making in patients with Bethesda III and IV thyroid nodules. Optimal management was observed in 67.5% of patients who underwent testing compared with 25.1% in those who did not. By supporting more accurate risk stratification and informing decisions about surgical extent, molecular testing was linked with fewer unnecessary procedures and a better alignment of treatment with pathology. The association was especially evident in Bethesda III nodules, where histologic behavior is less predictable. Developing cost-effective programs to expand access to molecular testing should be considered.

## Figures and Tables

**Table 1:** Surgical outcomes by molecular testing in all patients.

	Group	Total Patients (n)	Optimal Surgery (n, %)	Suboptimal Surgery (n, %)	*p*-Value
1	Molecular testing	203	137 (67.5%)	66 (32.5%)	*p* < 0.001
2	No molecular testing	207	52 (25.1%)	155 (74.9%)	

**Table 2:** Bethesda III surgical outcomes by molecular testing.

Group	Total Patients (n)	Optimal Surgery (n, %)	Suboptimal Surgery (n, %)	Total Thyroidectomies (n)	Hemi-Thyroidectomies (n)	Benign Hemi-Thyroidectomies (n)	*p*-Value
No molecular testing	129	27 (20.9%)	102 (79.1%)	30	72	64 (88.9%)	*p* < 0.001
Molecular testing	117	72 (61.5%)	45 (38.5%)	4	41	36 (87.8%)	

**Table 3:** Bethesda IV surgical outcomes by molecular testing.

Group	Total Patients (n)	Optimal Surgery (n, %)	Suboptimal Surgery (n, %)	Total Thyroidectomies (n)	Hemi-Thyroidectomies(n)	Benign Hemi-Thyroidectomies (n)	*p*-Value
No molecular testing	78	25 (32.1%)	53 (67.9%)	14	39	30 (76.9%)	*p* < 0.001
Molecular testing	86	65 (75.6%)	21 (24.4%)	1	20	14 (70.0%)	

## Data Availability

The data presented in this study are available on request from the corresponding author. The data are not publicly available due to privacy and ethical restrictions.
